# Overexpression of MMP14 is associated with poor prognosis and immune cell infiltration in colon cancer

**DOI:** 10.3389/fonc.2025.1564375

**Published:** 2025-04-25

**Authors:** Na Li, Nan Zhang, Guanghui Wang

**Affiliations:** ^1^ Department of Clinical Laboratory, The First Affiliated Hospital of Xi’an Jiao Tong University, Xi’an, Shaanxi, China; ^2^ Department of General Surgery, The First Affiliated Hospital of Xi’an Jiao Tong University, Xi’an, Shaanxi, China

**Keywords:** MMP14, prognosis, immune cell infiltration, CRC, machine learning, RNA-seq

## Abstract

**Introduction:**

Colorectal cancer (CRC) poses a significant risk of recurrence and distant metastases. This study investigated the regulatory role of Matrix metalloproteinase-14 (*MMP14*) in immune function and its impact on CRC prognosis.

**Methods:**

we performed transcriptome sequencing on tumor and adjacent non-cancerous samples from four pairs of patients diagnosed with colorectal cancer. Single-cell transcriptome data were analyzed to explore MMP14 expression and immune microenvironment changes. mRNA expression profiles and clinical data were retrieved from public databases (TCGA and GEO). The association between MMP14 and pathways as well as immune regulators was analyzed. Co-expression genes of MMP14 relevant to prognosis were identified. A prognostic model was then constructed. MMP14 expression was examined using real-time fluorescence quantification PCR (qRT-PCR) and Western blotting (WB). Immunofluorescence was utilized to demonstrate MMP14 expression in colon cancer tissues, while Hematoxylin and eosin (HE) staining was employed to observe the histology of normal tissue and colon cancer tissue.

**Results:**

Machine learning identified MMP14 as a candidate gene. MMP14 was overexpressed in CRC tissues and COLO205 cells. Single-cell transcriptome analysis revealed that MMP14 was highly expressed in fibrocyte cells within the liver metastasis group. Increased MMP14 levels correlated with poor overall survival (OS), progression-free survival (PFS), and advanced TNM stages. Functional assays indicated that silencing MMP14 in COLO205 cells enhanced apoptosis and upregulated the expression of the immune-related cytokine IL-1β. Furthermore, MMP14 exhibited significant correlations with immunomodulators, particularly immunostimulants and immunosuppressants, and was associated with immune cell infiltration within tumor tissues. Additionally, by utilizing co-expressed genes of MMP14 and conducting Cox regression analysis, we developed a risk prediction model comprising three genes (LIMK1, SPOCK3, SLC2A3). The risk scores derived from this model were found to correlate with OS and PFS.

**Discussion:**

MMP14 plays a crucial role in CRC progression. Its overexpression is related to poor prognosis and immune cell infiltration. The prognostic model based on MMP14 co-expression genes may help predict CRC prognosis. However, further studies are needed to validate these findings, such as more *in-vitro* and *in-vivo* experiments. In conclusion, MMP14 can serve as a biomarker for evaluating CRC prognosis and immune cell infiltration.

## 1 Introduction

Colorectal cancer (CRC) is a prevalent gastrointestinal tumor, ranking the third in cancer incidence (1–3). Notably, CRC incidence in China is rising, with an annual rise of 3.9% (4). Liver metastases manifest in approximately 43% of CRC-diagnosed patients, while 25% concurrently exhibit lung metastases. The primary predictor of survival remains the disease stage at diagnosis, with 5-year relative survival rates ranging from 91% to 14% for localized diseases (5). Therefore, it is imperative to undertake effective measures to investigate the underlying biological mechanisms governing CRC development, progression, and metastasis. Prognostic markers hold promise as they can complement clinicopathological diagnosis and potentially improve patient survival rates (6). Consequently, exploring the molecular mechanism of CRC is crucial for identifying effective treatment options.

Solid tumors comprise tumor cells, diverse non-tumor cells, and the extracellular matrix (ECM). For metastasis to occur, tumor cells acquire the capability to move and detach from the primary tumor, effectively overcoming the extracellular matrix barrier (7). Matrix metalloproteinases (MMPs), the key enzymes, are essential for ECM degradation and play critical roles in normal physiological processes and cancer-related activities, including migration and proliferation (8).

*MMP14*, a transmembrane protein, is a pivotal member of the MMP family and significantly enhances the metastatic potential of tumor cells by activating pro-MMP2 (9). A growing body of evidence indicates a substantial upregulation of *MMP14* expression across various tumor types, with an established impact on cellular migration, inflammation, and angiogenesis (10, 11). In this study, we examined the augmented levels of *MMP14* expression in colon tumor tissues compared to normal tissues. Furthermore, a positive correlation was observed between increasing *MMP14* expression and unfavorable prognosis outcomes. These findings underscore the critical need to investigate the mechanism underlying the oncogenic role of *MMP14* in CRC.

## 2 Methods

### 2.1 Data collection

We obtained distinct cohorts of Colorectal cancer patients from The Cancer Genome Atlas(TCGA) dataset, comprising RNA-seq data for 487 patients (446 tumor samples and 41 normal samples), and the GSE39582 dataset comprising microarray data for 566 tumor and 19 normal tissues using Illumina BeadArrays. The single-cell RNA sequencing (scRNA-seq) dataset GSE225857 was acquired from the Gene Expression Omnibus (GEO, http://www.ncbi.nlm.nih.gov/geo/), and it includes data from different tissues of six colorectal cancer (CRC) patients. Additionally, to further examine *MMP14* protein expression levels in CRC, we examined the Human Protein Atlas (HPA) database, focusing on *MMP14* protein expression in various malignancies. (www.proteinatlas.org/).

### 2.2 Survival analysis

Based on *MMP14* co-expression genes, we developed a prognostic model through Cox regression analysis. Briefly, this model utilizes a risk score index: the risk score can be calculated as the sum of A1 multiplied by X1, A2 multiplied by X2, and so on until Ai multiplied by Xi. A comprehensive analysis was conducted to evaluate the correlation between specific genes and clinical outcomes in CRC patients. Various statistical techniques were employed, including Kaplan-Meier assays, log-rank tests, and univariate and multivariate Cox regression analysis. Moreover, the prognostic assessment of risk scores was determined using receiver operating characteristic (ROC) curves and the survival ROC package (12).

### 2.3 Immune cell infiltration assay

Using 547 TAG-expressing data, the CIBERSORT algorithm, a deconvolution technique, was employed to assess the composition of immune cells in the samples (13). To determine the relative abundances of the 22 immune cell types in CRC, corrected transcriptome data were analyzed using the CIBERSORT R software tool. The threshold for statistical significance was set at *p* < 0.05.

### 2.4 Gene enrichment analysis

To identify *MMP14* co-expression genes, we utilized the STRING and GEPIA2 databases, setting specific parameters in the STRING database. Additionally, we used the gene detection tool Module in GEPIA 2 to identify *MMP14* co-expression genes in CRC, selecting a Pearson correlation coefficient (PCC) threshold of ≥ 0.74. Subsequently, the *MMP14* co-expression genes were merged with the STRING database and GEPIA2 database. Gene enrichment analysis was then conducted using the R 4.0.5 program, employing cluster Profiler, Enrichment plot, and ggplot2 packages.

### 2.5 Analysis of variations in the *MMP14* gene set using GSVA in CRC

To examine the role of *MMP14* in CRC, we employed Clinical Bioinformatics (14), facilitating a comprehensive analysis of tumor-infiltrating immune cells (TIICs). Furthermore, we utilized TIMER2.0, introduced by Li et al., enabling users to analyze immune cell presence in various cancer types through an interactive platform accessible at https://timer.cistrome.org/ (15).

### 2.6 The relationship between *MMP14* and tumor immune cell infiltration

To assess the correlation between *MMP14* and immune cell infiltration, we utilized TIMER and TIMER2.0. As suggested by Li et al., TIMER2.0 is an interactive digital platform that allows users to comprehensively examine tumor-infiltrating immune cells (TIICs) across various malignancies (https://timer.cistrome.org/) (16).

### 2.7 Tumor-immune system interactions and drug bank

The online platform TISIDB (http://cis.hku.hk/TISIDB/index.php) integrates multiple heterogeneous data sources to explore the interplay between the immune system and tumors. This database holds promise in predicting immunotherapy responses, identifying novel immunotherapy targets, and elucidating the relationship between immune cells and malignancies. It is poised to become a valuable tool for studying and treating cancer (17).

### 2.8 Cell culture

The COLO205 cell line was procured from Saibai Kang Biotechnology Co, Ltd (Cat No. iCell-h045; Shanghai, China). Cells were cultured in Roswell Park Memorial Institute-1640 (RPMI-1640) medium supplemented with 10% fetal bovine serum (Gibco, Carlsbad, CA, USA) and 1% penicillin-streptomycin (Cat No. C0222, Beyotime, Shanghai, China) at 37°C with 5.0% CO_2_. Routine checks for mycoplasma contamination were performed during cultivation. To silence the *MMP14* genes, a specific vector was constructed by Gene Pharma. Transfection of COLO205 cells with either the *MMP14* gene silencing vector or a negative control was performed at a concentration of 50 nM using Lipofectamine 2000 (Cat No. 11668019, Invitrogen, USA).

### 2.9 Human specimens

This study comprises three pairs of samples, including 3 normal tissues and 3 colon cancer tissues. All samples were from patients who underwent surgery at The First Affiliated Hospital of Xi’an Jiaotong University between July 1, 2023, and July 30, 2023. Ethical approval for this project was granted under the reference number XJTU1AF2023LSK-349. All procedures were conducted in compliance with the applicable rules and regulations. The Ethics Committee of The First Affiliated Hospital of Xi’an Jiaotong University approved the study and experimental procedures and provided from patient informed consent.

### 2.10 Western blot

To evaluate *MMP14* expression, Western blot analysis was performed. Initially, total proteins were extracted using the ProteoPrep Total Extraction Sample Kit (PROTTOTS, Sigma-Aldrich, Shanghai, China), following the manufacturer’s instructions. Subsequently, the proteins were transferred onto polyvinylidene fluoride membranes manufactured by Bio-Rad in Hercules, California, USA. To reduce non-specific binding, the membranes were blocked with a blocking solution. Primary antibodies specific to *MMP14* (1/1000 dilution; Cat No. 29111-1-AP; Proteintech, Wuhan, China) were then applied for detection. Glyceraldehyde-phosphate dehydrogenase (GAPDH; dilution: 1/10000; Cat No. 60004-1-Lg; Proteintech, Wuhan, China) was used as an internal reference for normalization. Subsequently, the membranes were incubated with goat anti-mouse secondary antibodies (dilution 1/1000; Cat No. A0216, Beyotime, Shanghai, China) and goat anti-rabbit secondary antibodies (dilution 1/1000; Cat No. A0208, Beyotime, Shanghai, China). Protein bands on the membranes were visualized using an enhanced chemiluminescent reagent (Cat No. P0018S, Beyotime, Shanghai, China). For quantification of WB results, densitometric analysis of WB bands was performed using the ImageJ analysis software.

### 2.11 Real-time fluorescence quantification PCR

qRT-PCR was conducted to assess *MMP14* expression levels. Total RNA was extracted from 50 mg of tumor tissue using Trizol reagent (Sigma-Aldrich, Cat No. T9424, MERCK, Shanghai, China). Subsequently, reverse transcription and qRT-PCR amplification were performed using the Transcriptor High Fidelity cDNA Synthesis Kit (Cat No. G3330-100, Service bio, Wuhan, China). To ensure accuracy, the experiments were conducted in triplicates. *GAPDH* served as an internal reference. Relative expression levels were normalized using the 2^−ΔΔct^ method. The primer sequences used are listed in **Table 1**.

**Table 1:** Primer sequences table for quantitative detection of target genes.

Target genes	Primer sequences (5’to3’)	Amplified fragment size (bp)
MMP14-F	CGAGGTGCCCTATGCCTAC	
MMP14-R	CTCGGCAGAGTCAAAGTGG	178
IL1B-F	GGCCCTAAACAGATGAAGTGC	
IL1B-R	TCGGAGATTCGTAGCTGGAT	80

### 2.12 Cell apoptosis detection

Cell apoptosis was assessed using the Annexin V-FITC/PI apoptosis detection kit (Catalog #E-CK-A211) from Ela Science Biotechnology Co., Ltd. in Wuhan, China, following the manufacturer’s instructions. Briefly, COLO205 cells (3×10^5 cells/well) were seeded onto a 6-well plate, harvested after 48 hours, and washed with phosphate-buffered saline. The cells were then resuspended in 100 μl of Annexin V binding buffer and stained following the manufacturer’s protocol. Stained cells were analyzed using a flow cytometer from BD Biosciences in the USA. Each experiment examined 10,000 cells to determine the proportion of PI-positive/negative and Annexin V-FITC-positive cells.

### 2.13 Immunofluorescence assay

Fresh CRC tissues were obtained for immunofluorescence analysis and fixed in an ice-cold 4% paraformaldehyde solution overnight. Following fixation, the tissues were infiltrated with a combination of 30% sucrose phosphate buffer for one day. Subsequently, all samples were snap-frozen in an optimal cutting temperature medium using dry ice and then sliced into 5 μm thick using a cryostat. Immunofluorescence staining was performed, primary and secondary antibodies were hybridized overnight at a 1/200 dilution (Cat. No. 29111-1-AP; Proteintech, Wuhan, China). Nuclei were counterstained with DAPI (Cat. No. C1002, Beyotime, Shanghai, China).

### 2.14 Hematoxylin and eosin staining

A human colorectal tissue specimen was embedded in paraffin, mounted, and scanned using a pathological section scanner after fixation with 4% paraformaldehyde (pH 7.4), gradual dehydration, and sectioning into 4 μm sections. Subsequently, the tissue sections were stained with H&E.

### 2.15 Statistical analysis

R software (v4.3.1) was employed for all statistical analyses. Continuous data with a normal distribution were analyzed using Student’s *t*-test, while categorical variables were compared using the Pearson chi-square test. Patient overall survival (OS) and progression-free survival (PFS) across different subgroups were evaluated using the Kaplan-Meier technique with a two-sided log-rank test. Statistical significance was defined as a *p*-value less than 0.05, with significance levels indicated as *p < 0.05, **p < 0.01, ***p < 0.001, and ****p < 0.0001.

### 2.16 RNA-seq sample collection

This study involved the selection of biological samples from four patients. Each patient provided two samples: one of normal tissue, serving as the control group, and one of cancer tissue, constituting the experimental group. The samples were rapidly frozen and preserved immediately after collection to prevent biodegradation, thereby ensuring the reliability and accuracy of subsequent analyses. The selection of samples was based on clinical diagnoses and received approval from the ethics committee.

### 2.17 RNA-sequencing

The concentration of RNA samples was measured by using a Nanodrop2000 and the integrity of the RNA was confirmed using the Agilent 2100 BioAnalyzer. The Ribo-ZeroTM kit (Epicentre, Beijing, China) was used to remove ribosomal RNA. cDNA was synthesized from the RNA using random hexamer primers and the QIAGEN Reverse Transcription Kit following the manufacturer’s instructions and submitted to a commercial company (Novogene Co., Beijing, China) for transcriptome sequencing analysis. The Illumina Hiseq sequencing platform was used for transcriptome sequencing. An Illumina PE library was constructed for 2 × 150 bp sequencing, and the obtained sequencing data were subjected to quality control.

### 2.18 RNA-seq data analysis

The sequencing data obtained were subjected to quality control using fastp (version 0.23.2). Adapters and low-quality bases were removed, and low-quality data were filtered based on Q value to ensure the integrity of the analysis results. Following quality control, the cleaned sequences were saved in FASTQ format. The processed sequences were then aligned to the reference genome (human genome GRCh38) using HISAT2 (version 2.2.1). Upon completion of the alignment, the output file was saved in SAM format, providing a foundation for subsequent data processing. Samtools (version 1.13) was utilized to convert SAM files to BAM format and to sort them. This step not only enhances the efficiency of the subsequent analyses but also accelerates data access by indexing BAM files, facilitating the rapid retrieval of the required sequence information. Gene read counting from the sequenced BAM files was conducted using featureCounts (version 2.0.3). By integrating gene annotation files, the accuracy and reliability of the counting process were ensured. After obtaining the gene expression levels, gene annotation was performed, low-expression genes were filtered out, and differential expression analysis was executed using the DESeq2 R package. Initially, a DESeqDataSet object containing both normal and cancer samples was constructed. Subsequently, a normalization method was applied, and the negative binomial distribution model was employed for differential analysis. To identify significantly differentially expressed genes, the thresholds were set to log2 Fold Change (logFC) > 1 and P < 0.05. Ultimately, a total of 896 down-regulated genes and 685 up-regulated genes were identified. Following the identification of significantly differentially expressed genes, Gene Ontology (GO) and Kyoto Encyclopedia of Genes and Genomes (KEGG) enrichment analyses were performed to assess whether the differential gene sets were significantly enriched in specific biological pathways or functional categories.

### 2.19 Machine learning to screen differential genes

The differences genes were further analyzed using Support Vector Machines Recursive Feature Elimination (SVM-RFE), Least Absolute Shrinkage and Selection Operator (LASSO), and Random Forest (RF) algorithms. SVM-RFE iteratively screens genes to identify those that exert the greatest influence on the model. LASSO facilitates sparse selection of genes through the application of penalty coefficients, while the RF algorithm is adept at processing high-dimensional data and effectively identifying key gene characteristics. However, due to limitations in sample size, only four genes (ENTREP1, KIAA0513, PCSK2, PITPNM3, MMP14) were successfully identified through LASSO regression.

### 2.20 Single cell transcriptome data analysis

Immune and non-immune cell data were extracted from the single-cell RNA sequencing dataset GSE217517. The expression levels of MMP14 in these two groups were analyzed preliminarily. To ensure data quality, quality control was performed. Specifically, low-quality cells (cells with fewer than 200 expressed features) were removed. The data were then normalized and scaled using the NormalizeData and ScaleData functions from the ‘Seurat’ package. We selected the top 2000 most variable genes using the FindVariableFeatures function. Next, principal component analysis (PCA) was performed on the data using the RunPCA function in Seurat, followed by batch effect correction with the Harmony package. Subsequently, cell clustering was performed using the FindClusters function with a resolution of 0.2 and the Louvain clustering algorithm to identify cell subpopulations. To further explore the distribution and structure of the cells, t-distributed Stochastic Neighbor Embedding (t-SNE) was used for nonlinear dimensionality reduction, facilitating the visualization of relationships between different cell populations. Based on the t-SNE visualization, we observed the distribution of different cell subpopulations. We then annotated the cell subpopulations using common cell marker genes for classification. By comparing gene expression features across the different populations, we further analyzed the differences between immune and non-immune cells, with a particular focus on the expression of MMP14 in the various subpopulations.

## 3 Results

### 3.1 Machine learning screening of transcriptome candidate genes

**Figure 1:**
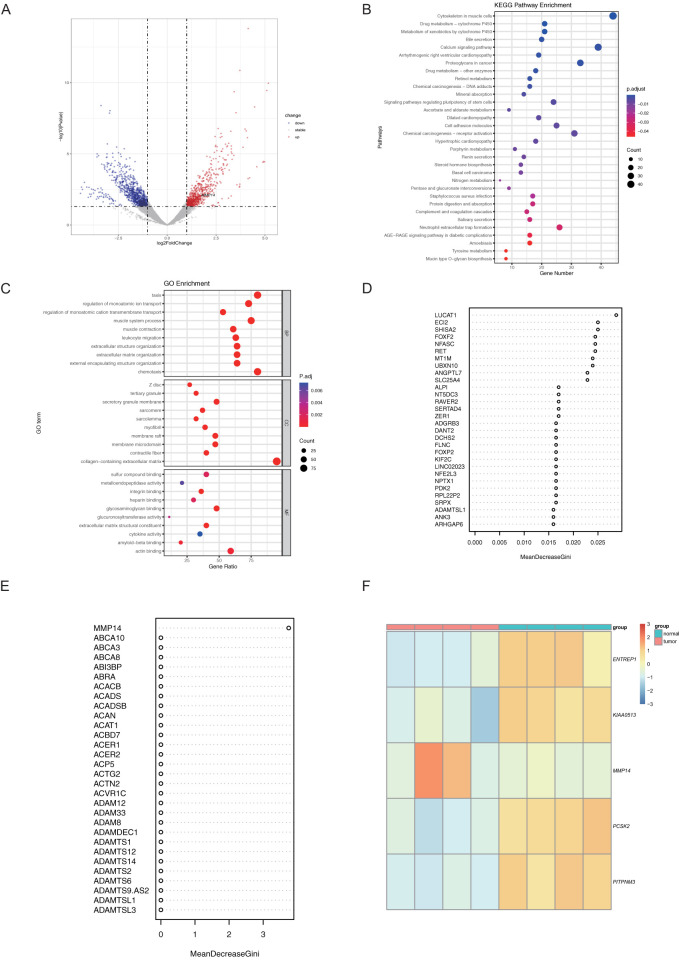
Transcriptome data were analyzed using machine learning to identify candidate genes. **(A)** Presents a volcano plot illustrating differential transcriptome expression; **(B)** The results of Gene Ontology (GO) enrichment analysis for significantly differentially expressed genes; **(C)** The results of Kyoto Encyclopedia of Genes and Genomes (KEGG) enrichment analysis for these genes; **(D, E)** Candidate genes were identified using the Random Forest (RF) method in **(D)** and the Least Absolute Shrinkage and Selection Operator (LASSO) method in **(E)**; **(F)** Heatmap depicting the differential expression of the candidate genes.

Initially, we conducted a transcriptomic differential analysis on cancerous and paracancerous samples, revealing that the number of up-regulated and down-regulated significant differential genes was comparable between the two groups (**Figure 1A**). Furthermore, the Gene Ontology (GO) and Kyoto Encyclopedia of Genes and Genomes (KEGG) enrichment analyses did not identify tumor-related pathways (**Figures 1B, C**). Consequently, we employed machine learning techniques to analyze the significantly different genes between the two groups. Specifically, we utilized Support Vector Machines with Recursive Feature Elimination (SVM-RFE), Least Absolute Shrinkage and Selection Operator (LASSO), and Random Forest (RF) algorithms to further screen for key genes among the differential genes. Our results identified four related differentially expressed genes through LASSO: ENTREP1, KIAA0513, PCSK2, and PITPNM3 (**Figure 1D**). Additionally, the RF algorithm highlighted MMP14 (**Figures 1E, F**). Based on existing literature on colorectal cancer, we selected MMP14 as the final candidate gene for subsequent studies. To investigate variations in *MMP14* expression between normal and tumor tissues, mRNA levels of *MMP14* were examined across different cancer types and their respective normal tissues utilizing the TIMER database (www.timer.cistrome.org/) (18, 19). Our analysis revealed a notable increase in MMP14 mRNA levels across various cancer types, including BLCA and COAD, among others (**Supplementary Figure S1A**). Conversely, *MMP14* expression levels decreased in KICH, PRAD, and UCEC cancers. Immunohistochemistry (IHC) analysis was performed on normal and tumor tissues from CRC patients to assess MMP14 protein expression. The findings illustrated in **Supplementary Figure S1B** demonstrate a significant elevation in MMP14 protein expression in CRC compared to normal tissues.

### 3.2 Relationship between MMP14 and immune microenvironment in colorectal cancer

**Figure 2:**
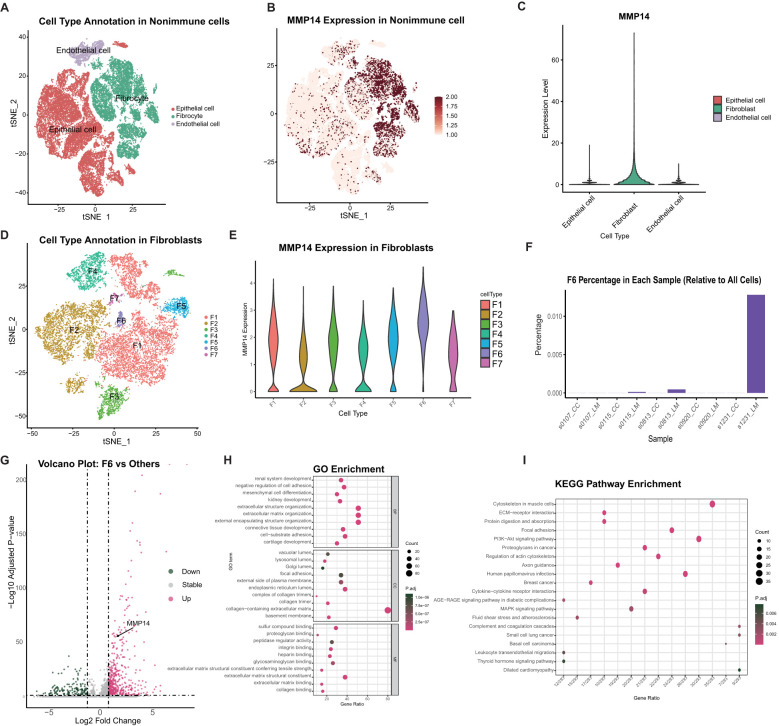
Single-cell transcriptome analysis of the relationship between MMP14 and colorectal cancer. **(A)** CD45 negative non-immune cell annotated tSNE; **(B)** Expression of MMP14 in different cell subsets of tSNE; **(C)** The expression of MMP14 among different cell subsets; **(D)** Fibroblasts subgroup subdivision; **(E)** The expression of MMP14 in different subpopulations after Fibroblasts subgroups; **(F)** Fib6 ratio among different samples; **(G)** Fib6 and other Fib difference analysis volcano map; **(H)** GO enrichment analysis of differential genes; **(I)** KEGG enrichment analysis of differential genes.

**Figure 3:**
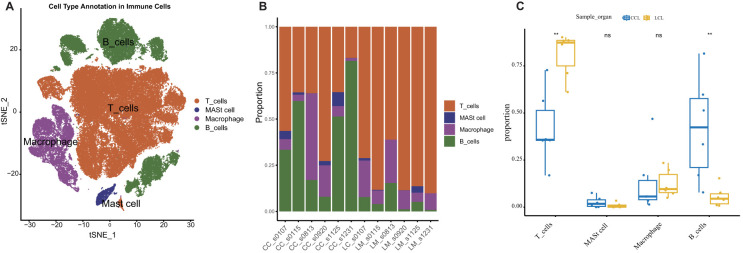
Single-cell transcriptome analysis of the relationship between MMP14 and colorectal cancer. **(A)** CD45-positive immune cell annotated tSNE; **(B)** Histogram of the proportional accumulation of immune cells between different samples; **(C)** A 50-point bitmap of the significant difference of immune cells between the two groups. Statistical significance is denoted as **P < 0.01. ns means no significant difference, p greater than 0.05.

To further elucidate the relationship between high MMP14 gene expression and colorectal cancer, we utilized single-cell transcriptome data from public databases for analysis (**Figure 2A**). Our findings indicated that MMP14 is predominantly expressed in non-immune cells. A more detailed examination of these non-immune cells revealed that MMP14 expression was particularly high in fibrocyte cells (**Figures 2B, C**). Consequently, we subdivided the fibrocyte population, resulting in the identification of six distinct cell subpopulations (**Figure 2D**). Among these, the Fib6 subpopulation exhibited the highest expression of MMP14 (**Figure 2E**). Upon analyzing samples derived from the Fib6 subpopulation, we discovered that this population primarily originates from liver metastasis samples (LM) (**Figure 2F**). We subsequently conducted differential analysis on the Fib6 subpopulation to investigate the functional role of fibrocyte cells with elevated MMP14 expression (**Figure 2G**). The results of Gene Ontology (GO) enrichment analysis and Kyoto Encyclopedia of Genes and Genomes (KEGG) enrichment analysis of the differentially expressed genes indicated that these differences are associated with pathways closely related to tumorigenesis, including the PI3K-Akt, MAPK, and proteoglycans in cancer pathways (**Figures 2H, I**). Subsequently, we conducted an analysis of immune cells using single-cell sequencing (**Figure 3A**). The results indicated that immune cells could be primarily categorized into four major groups. Notably, significant differences were observed in immune cell populations, particularly T cells and B cells, between the MMP14 high expression group (LM) and the low expression group (CC) (**Figures 3B, C**). These findings suggest that elevated MMP14 expression is associated with the progression of colorectal cancer and may influence the tumor-related immune microenvironment.

### 3.3 Association of *MMP14* overexpression with prognosis

The impact of *MMP14* on colorectal adenocarcinoma (COAD) was investigated by analyzing OS and PFS in TCGA-COAD cohorts with varying expression levels. Our analysis revealed significant associations between *MMP14* expression and both OS and PFS (**Supplementary Figure S2**). Specifically, elevated *MMP14* levels were correlated with unfavorable outcomes, including OS (*p* = 0.035) and PFS (*p* = 0.017). These findings were further validated using the GSE39582 dataset, confirming a significant impact of the *MMP14* gene on prognosis (*p* < 0.001). Results of correlation analysis between mmp14 expression and poor prognosis underscores the potential of *MMP14* as a prognostic biomarker for COAD. The results from external validation further highlight the importance of *MMP14* in determining patient outcomes, suggesting its prospective utility in clinical practice for predicting COAD prognosis.

### 3.4 Association between *MMP14* expression and clinical characteristics

Subsequently, we investigated the correlation between *MMP14* expression and pathological findings. The characteristics of the patients were classified into two groups, revealing notable correlations between *MMP14* mRNA levels and patient age (*p* = 0.046), progression to advanced TNM stage (*p* = 0.025), and N stage (*p* = 0.0011). Notably, no significant association was observed between *MMP14* concentrations and various clinicopathological variables, including sex (*p* = 0.99), tumor stage (*p* = 0.083), and metastasis stage (*p* = 0.51), as demonstrated in **Supplementary Figures S3A–F**.

### 3.5 Analysis of immune cell infiltration

Initially, a histogram (**Supplementary Figure S4A**) showed the distribution of 22 different immune cell types in each sample. The hues in the histogram represent the proportion of various immune cells in each sample. Subsequently, an evaluation was conducted to assess the correlation across 22 distinct types of immune cells in CRC tissues. **Supplementary Figure S4B** shows negative correlation in blue and positive correlation in red. An example is the strong connection between active mast cells and neutrophil cells. Macrophages M0 and resting NK cells positively correlated with activated mast cells. Conversely, resting mast cells and plasma cells negatively correlated with macrophage M0 cells. Additionally, the Wilcoxon test was employed to elucidate notably distinct immunological cell infiltrates in CRC.

The results of the Wilcoxon test are shown in **Supplementary Figure S4C** using a violin plot, which highlights 14 different immune cells (*p* < 0.05). Blue samples represent normal tissues, while red ones denote CRC samples. Plasma cells, dendritic cells in a resting state, gamma delta T cells, activated NK cells, macrophages M2, eosinophils, and resting mast cells exhibited a significant decrease in CRC compared to normal tissues. Conversely, resting CD4 memory T cells, follicular helper T cells, resting NK cells, macrophages M0, macrophages M1, activated mast cells, and neutrophils increased in CRC samples.

### 3.6 MMP14 expression levels and immune cell infiltration levels

We examined the potential relationship between *MMP14* and tumor-infiltrating immune cells (TIICs) to elucidate the CRC microenvironment and its potential impact on CRC heterogeneity and prognosis. MMP14 expression was positively correlated with the presence of CD8+ T cells (r = 0.226, *p* = 4.43^e-06^), CD4+ T cell infiltration (r = 0.501, *p* = 5.75^e-27^), macrophage infiltration (r = 0.512, *p* = 2.04^e-28^), neutrophil infiltration (r = 0.509, *p*-value = 7.36^e-28^), dendritic cell infiltration (r = 0.546, *p* = 1.50^e-32^), and tumor purity (r = -0.343, *p* = 1.11^e-12^). **Supplementary Figure S5A** illustrates no significant association with B cell infiltration (r = 0.035, *p* = 4.86^e-01^).

Furthermore, we analyzed several differential immune cells, including neutrophils, dendritic cells, macrophages (M0, M1, M2), and other immune cells using different calculation algorithms such as CIBERSORT-ABS, MCPCOUNTER, XCELL, EPIC, and QUANTISEQ. Our results indicate that *MMP14* expression is significantly positively associated with the previously highlighted immune cells (**Supplementary Figures S5B-G**).

### 3.7 Association of immunomodulators with *MMP14* expression

Immunomodulators are compounds that influence the operation of the immune system. We analyzed the relationship between *MMP14* expression and immunomodulators using immunoinhibitors and immunostimulators. Through the TISIDB database, we identified *MMP14*-related immunomodulators, and the heatmaps of immunopotentiators and immunosuppressants are shown in **Supplementary Figures S6A, B**. Our results indicated a significant connection between MMP14 and immunoinhibitors, such as TGFB1 (rho = 0.575), CSF1R (rho = 0.51), PDCD1LG2 (rho = 0.424), HAVCR2 (rho = 0.446) (**Supplementary Figures S6C-F**). The expression of ITGAL was also closely associated with immunostimulators, including TNFSF4 (rho = 0.629), CD86 (rho = 0.443), ENTPD1 (rho = 0.459), CD276 (rho = 0.436), TNFRSF8 (rho = 0.438), CXCL12 (rho = 0.412), and CXCR4 (rho = 0.405) (**Supplementary Figures S6J-M**). These findings suggest that ITGAL regulates tumor immune escape and is directly involved in immune interaction modulation.

### 3.8 Enrichment analysis of *MMP14* co-expression genes

We utilized the TCGA datasets to analyze *MMP14* co-expression, revealing a significant number of *MMP14* co-expression genes. The genes and their correlations with *MMP14* are presented in **Table 2**. Subsequently, we conducted gene ontology (GO) and Kyoto Encyclopedia of Genes and Genomes (KEGG) analyses on the co-expression genes (**Supplementary Figure S7A**). **Supplementary Figure S7B** displays the top 30 KEGG pathways, with proteoglycans in cancer ranking first. Additionally, the co-expressed genes were found to be associated with well-known signaling pathways such as PI3K-Akt, Rap1, relaxin, TGF-β, and Hedgehog. These pathways are strongly associated with the development of various malignancies as they play essential roles in controlling numerous biological functions, including metabolism and cell proliferation.

**Table 2:** Table of the genes significantly associated with MMP14 analyzed by TCGA database.

Query	Gene	cor	pvalue
MMP14	ACAN	0.40604623	3.92E-19
MMP14	ACTB	0.29177504	3.35E-10
MMP14	ADAM10	0.09938249	0.035893041
MMP14	ADI1	-0.2700313	6.84E-09
MMP14	AGA	-0.1071759	0.023599377
MMP14	BCAR1	0.30897082	2.55E-11
MMP14	C1QBP	-0.2865908	7.04E-10
MMP14	CAV1	0.59718547	1.90E-44
MMP14	CCL7	0.43484261	5.34E-22
MMP14	CD9	-0.046238	0.329923383
MMP14	CILP	0.58740584	1.01E-42
MMP14	CILP2	0.34586268	5.61E-14
MMP14	CLDN1	0.09498936	0.044967458
MMP14	DLL1	0.28540506	8.32E-10
MMP14	ENG	0.62168206	4.78E-49
MMP14	F12	-0.1750552	0.000202968
MMP14	FGFR2	0.17356976	0.000230255
MMP14	FGFR4	-0.1576008	0.000838142
MMP14	FURIN	0.31725392	6.93E-12
MMP14	GGCT	-0.3545582	1.17E-14
MMP14	GMDS	-0.1843119	9.04E-05
MMP14	GOLGB1	0.14097639	0.00284693
MMP14	GORASP2	0.10068204	0.033527248
MMP14	HSPA2	0.35532375	1.02E-14
MMP14	KISS1	0.00299906	0.949640102
MMP14	LIMK1	0.31907051	5.18E-12
MMP14	LRP1	0.39645822	3.08E-18
MMP14	MMP13	0.50466958	3.39E-30
MMP14	MMP3	0.20746578	1.00E-05
MMP14	NPR1	0.61489111	9.88E-48
MMP14	RDX	0.32885553	1.04E-12
MMP14	SDC1	0.00371818	0.937587037
MMP14	SPOCK3	0.35148534	2.05E-14
MMP14	TGOLN2	0.06464805	0.17292092
MMP14	TIMP1	0.51864402	4.48E-32
MMP14	TIMP4	-0.1202474	0.011035513
MMP14	TSPAN12	-0.2357949	4.73E-07
MMP14	UBASH3B	0.35495407	1.09E-14
MMP14	UBE4A	0.10924046	0.021029577
MMP14	UBE4B	0.08394223	0.076576759
MMP14	VPS35	-0.0538017	0.256855044
MMP14	COL6A2	0.84541984	5.20E-123
MMP14	COL6A3	0.85367638	7.07E-128
MMP14	ADAMTS12	0.79275713	1.65E-97
MMP14	COL1A2	0.87842319	1.79E-144
MMP14	CERCAM	0.81934101	2.44E-109
MMP14	COL5A1	0.8679631	4.76E-137
MMP14	ADAMTS2	0.82281571	4.98E-111
MMP14	COL5A2	0.82528054	2.99E-112
MMP14	ARSI	0.81581722	1.16E-107
MMP14	NTM	0.82409464	1.16E-111
MMP14	PDGFRB	0.8575534	2.88E-130
MMP14	COL1A1	0.879092	5.69E-145
MMP14	MRC2	0.89039538	7.33E-154
MMP14	SCARF2	0.7204412	1.41E-72
MMP14	PRRX1	0.72549165	4.68E-74
MMP14	GPR68	0.77550792	1.01E-90
MMP14	RCN3	0.71997427	1.92E-72
MMP14	TIMP2	0.83293872	3.58E-116
MMP14	SPARC	0.78026029	1.57E-92
MMP14	SULF1	0.79172964	4.37E-97
MMP14	COL6A1	0.83464939	4.48E-117
MMP14	ANTXR1	0.77405648	3.53E-90
MMP14	MMP2	0.82440938	8.13E-112
MMP14	COL12A1	0.78294262	1.43E-93
MMP14	ZNF469	0.833333	2.22E-116
MMP14	ANGPTL2	0.83744386	1.42E-118
MMP14	COL3A1	0.84112882	1.36E-120
MMP14	BMP1	0.69872649	1.41E-66
MMP14	LOXL2	0.8031877	6.21E-102
MMP14	ADAMTS7	0.74768632	5.77E-81
MMP14	RAB31	0.75042406	7.22E-82
MMP14	LGALS1	0.67377805	2.57E-60
MMP14	SPON2	0.76877708	3.09E-88
MMP14	THY1	0.77094967	4.97E-89
MMP14	SNAI2	0.67992149	8.42E-62
MMP14	SYDE1	0.7829431	1.43E-93
MMP14	INHBA	0.73223505	4.41E-76
MMP14	PCOLCE	0.76452603	1.04E-86
MMP14	CTHRC1	0.7734189	6.09E-90
MMP14	GPC6	0.65725126	1.69E-56
MMP14	RAI14	0.5975241	1.65E-44
MMP14	CMTM3	0.7384961	5.10E-78
MMP14	VASN	0.74333554	1.49E-79
MMP14	COL18A1	0.76666102	1.79E-87
MMP14	CLEC11A	0.59447216	5.80E-44
MMP14	FBN1	0.79580363	8.97E-99
MMP14	EVC	0.79772814	1.39E-99
MMP14	AXL	0.71790475	7.56E-72
MMP14	ISLR	0.79285645	1.51E-97
MMP14	EFS	0.77454136	2.32E-90
MMP14	P4HA3	0.80665023	1.84E-103
MMP14	HHIPL1	0.78388285	6.13E-94
MMP14	GPR176	0.75124838	3.84E-82
MMP14	MXRA8	0.80082581	6.58E-101
MMP14	PDPN	0.71379555	1.11E-70
MMP14	AEBP1	0.86846594	2.16E-137
MMP14	COL11A1	0.76172386	1.01E-85
MMP14	VIM	0.69465954	1.64E-65
MMP14	EMILIN1	0.82498938	4.18E-112
MMP14	ST6GALNAC5	0.71780926	8.05E-72
MMP14	NID2	0.75110862	4.28E-82
MMP14	TNFAIP6	0.66207435	1.38E-57
MMP14	BGN	0.85533867	6.82E-129
MMP14	SERPINH1	0.66548596	2.27E-58
MMP14	MXRA5	0.75566124	1.26E-83
MMP14	PDGFB	0.71729573	1.13E-71
MMP14	VCAN	0.73461353	8.23E-77
MMP14	P3H1	0.71059554	8.68E-70
MMP14	VSTM4	0.76222229	6.77E-86
MMP14	EFEMP2	0.78814355	1.25E-95
MMP14	NOTCH3	0.76645349	2.13E-87
MMP14	POSTN	0.71790732	7.55E-72
MMP14	THBS2	0.8174595	1.94E-108
MMP14	GLT8D2	0.69329595	3.69E-65
MMP14	COL24A1	0.67254611	5.04E-60
MMP14	EMP3	0.65716613	1.77E-56
MMP14	COL10A1	0.7686569	3.42E-88
MMP14	LAMA4	0.69533362	1.09E-65
MMP14	CCDC8	0.82379412	1.64E-111
MMP14	PODNL1	0.64430211	1.14E-53
MMP14	C1QTNF6	0.66540047	2.37E-58
MMP14	GJA1	0.56762052	2.13E-39
MMP14	UBTD1	0.60947888	1.05E-46
MMP14	EHD2	0.81219033	5.68E-106
MMP14	DKK3	0.76464817	9.40E-87
MMP14	CD248	0.78975758	2.78E-96
MMP14	HTRA1	0.73308869	2.42E-76
MMP14	SGIP1	0.61088658	5.70E-47
MMP14	PRR16	0.67758699	3.12E-61
MMP14	PXDN	0.80602356	3.50E-103
MMP14	SPOCK1	0.78997948	2.26E-96
MMP14	SDC2	0.63632671	5.37E-52
MMP14	PLXDC2	0.66970643	2.36E-59
MMP14	LUM	0.70670633	1.02E-68
MMP14	ADAMTS14	0.64067041	6.67E-53
MMP14	ITGAV	0.4657585	2.14E-25
MMP14	MCC	0.66028059	3.52E-57
MMP14	AC112721.2	0.591078	2.31E-43
MMP14	CALU	0.45715375	2.05E-24
MMP14	TSHZ3	0.73715358	1.34E-77
MMP14	TENM4	0.77245138	1.39E-89
MMP14	CTSK	0.66966254	2.42E-59
MMP14	ITPRIP	0.6346093	1.21E-51
MMP14	GLI3	0.78305356	1.30E-93
MMP14	MAFB	0.6639236	5.19E-58
MMP14	ATP10A	0.77473593	1.97E-90
MMP14	GAS1	0.75033067	7.76E-82
MMP14	ELK3	0.56784071	1.96E-39
MMP14	KCND2	0.67117372	1.07E-59
MMP14	OLFML1	0.66131569	2.05E-57
MMP14	ZNF521	0.71415515	8.78E-71
MMP14	ITGB1	0.49517137	5.75E-29
MMP14	DCN	0.6613624	2.00E-57
MMP14	BICC1	0.59800529	1.35E-44
MMP14	FSTL1	0.70918522	2.13E-69
MMP14	COL8A1	0.76858007	3.64E-88
MMP14	LOX	0.69622487	6.40E-66
MMP14	GNAI2	0.67108632	1.12E-59
MMP14	DSEL	0.60299267	1.68E-45
MMP14	SH3PXD2B	0.59115783	2.24E-43
MMP14	ADGRA2	0.76026818	3.27E-85
MMP14	SGCD	0.66948602	2.66E-59
MMP14	FIBIN	0.6947944	1.51E-65
MMP14	NRP1	0.64541902	6.56E-54
MMP14	GFPT2	0.77521524	1.30E-90
MMP14	PDLIM2	0.35488719	1.10E-14
MMP14	FRMD6	0.72615694	2.97E-74
MMP14	ITGA11	0.78951167	3.50E-96
MMP14	BASP1	0.70907613	2.28E-69
MMP14	CSGALNACT2	0.60601503	4.64E-46
MMP14	CD276	0.49607465	4.41E-29
MMP14	GGT5	0.85011718	9.62E-126
MMP14	MFGE8	0.78929281	4.29E-96
MMP14	CLMP	0.69162794	9.89E-65
MMP14	ADAM12	0.77634517	4.88E-91
MMP14	CHSY3	0.64026482	8.12E-53
MMP14	RAB34	0.69552378	9.75E-66
MMP14	HIC1	0.57493631	1.34E-40
MMP14	FBXL7	0.76991399	1.19E-88
MMP14	NUAK1	0.70685349	9.29E-69
MMP14	UBE2QL1	0.63808888	2.31E-52
MMP14	PDGFC	0.62687562	4.48E-50
MMP14	PPP1R18	0.7570306	4.28E-84
MMP14	MSN	0.68838618	6.62E-64
MMP14	PKD2	0.58524163	2.40E-42
MMP14	C1R	0.79556239	1.13E-98
MMP14	LBH	0.68784682	9.06E-64
MMP14	NRP2	0.70437449	4.38E-68
MMP14	SPSB1	0.7114454	5.04E-70
MMP14	CYP7B1	0.56269545	1.32E-38
MMP14	COL8A2	0.75603783	9.35E-84
MMP14	FKBP7	0.45030234	1.18E-23
MMP14	ZNF532	0.70987624	1.37E-69
MMP14	MITF	0.65879447	7.61E-57
MMP14	GLI2	0.77682402	3.22E-91
MMP14	ADAMTS4	0.67408778	2.16E-60
MMP14	PLXDC1	0.60465448	8.29E-46
MMP14	TWIST2	0.68387241	8.95E-63
MMP14	CHST11	0.66656445	1.28E-58
MMP14	C1QTNF5	0.02080821	0.661200161
MMP14	ALPK2	0.64760461	2.23E-54
MMP14	BNC2	0.687327	1.22E-63
MMP14	MSC-AS1	0.56224444	1.55E-38
MMP14	TSPAN4	0.62375997	1.86E-49
MMP14	SERPINF1	0.73725208	1.25E-77
MMP14	CHST3	0.652714	1.72E-55
MMP14	ITGA5	0.79200761	3.36E-97
MMP14	LRRC15	0.65795073	1.18E-56
MMP14	FBLN2	0.77642967	4.54E-91
MMP14	MAP7D1	0.67958836	1.02E-61
MMP14	ENTPD1	0.50596535	2.29E-30
MMP14	COL5A3	0.68945617	3.54E-64
MMP14	MRAS	0.77288833	9.59E-90
MMP14	CHSY1	0.47945246	5.14E-27
MMP14	MYO5A	0.5937862	7.68E-44
MMP14	ARHGAP31	0.60469639	8.14E-46
MMP14	C1S	0.73165341	6.63E-76
MMP14	THBD	0.64750013	2.34E-54
MMP14	CNPY4	0.58869821	6.04E-43
MMP14	TCF4	0.59859772	1.06E-44
MMP14	COL4A2	0.7458684	2.26E-80
MMP14	HLX	0.59222613	1.45E-43
MMP14	SSC5D	0.83087341	4.28E-115
MMP14	IL1R1	0.65787944	1.22E-56
MMP14	FAM20C	0.7390124	3.51E-78
MMP14	LOXL3	0.59931644	7.83E-45
MMP14	SDK1	0.67149165	8.96E-60
MMP14	GREM1	0.70239714	1.49E-67
MMP14	LOXL1	0.65512955	5.02E-56
MMP14	GAS7	0.730943	1.09E-75
MMP14	CHST15	0.60977967	9.21E-47
MMP14	ZEB2	0.63752622	3.03E-52
MMP14	FZD1	0.64823009	1.63E-54
MMP14	MAP1A	0.76363749	2.15E-86
MMP14	CPXM1	0.69383552	2.67E-65
MMP14	GDF6	0.62508926	1.02E-49
MMP14	PPFIA2	0.63303779	2.55E-51
MMP14	PCDH7	0.66880526	3.84E-59
MMP14	SLC39A13	0.51460148	1.60E-31
MMP14	MAF	0.63325836	2.30E-51
MMP14	IKBIP	0.42460079	6.01E-21
MMP14	OLFML2B	0.81523686	2.18E-107
MMP14	MSC	0.70702115	8.36E-69
MMP14	CLIC4	0.4277442	2.88E-21
MMP14	PTGIR	0.66905906	3.35E-59
MMP14	MMP19	0.72331425	2.05E-73
MMP14	EVC2	0.74125694	6.85E-79
MMP14	LZTS1	0.70577439	1.83E-68
MMP14	TIMP3	0.70221607	1.67E-67
MMP14	KCNE4	0.7473501	7.44E-81
MMP14	QKI	0.53509913	2.11E-34
MMP14	SRGAP2	0.54527601	6.58E-36
MMP14	MMP16	0.59768729	1.54E-44
MMP14	LINC01561	0.67762971	3.04E-61
MMP14	GLIS2	0.78096152	8.43E-93
MMP14	C5AR1	0.58890555	5.56E-43
MMP14	ETS1	0.6141605	1.36E-47
MMP14	GNB4	0.55921819	4.69E-38
MMP14	PCDHGA12	0.65754076	1.46E-56
MMP14	CCIN	0.59173094	1.78E-43
MMP14	BMP8A	0.5812603	1.15E-41
MMP14	DCBLD1	0.32453946	2.13E-12
MMP14	COPZ2	0.71254319	2.49E-70
MMP14	RFX8	0.44594904	3.52E-23
MMP14	AC106786.1	0.63845468	1.94E-52
MMP14	CDK14	0.64559285	6.02E-54
MMP14	COLEC12	0.64102466	5.62E-53
MMP14	HSPG2	0.61821399	2.27E-48
MMP14	HSPA12B	0.67711287	4.06E-61
MMP14	ARHGEF17	0.73192597	5.48E-76
MMP14	SLC2A3	0.59485367	4.96E-44
MMP14	HS3ST3B1	0.47388363	2.39E-26
MMP14	BCL6B	0.67970717	9.50E-62
MMP14	TMEM26	0.38477915	3.46E-17

### 3.9 GSVA analysis

Moreover, the analysis of *MMP14*-related gene set variation analysis (GSVA) results revealed a positive correlation between *MMP14* expression and various biological processes, including tumor inflammation, epithelial-to-mesenchymal transition (EMT) markers, and extracellular matrix (ECM) degradation. Conversely, *MMP14* expression exhibited an inverse relationship with tumor proliferation, G2M checkpoint activity, and related processes. The associations were evaluated using distinct standards ranging from 0.00 to 1.0, indicating very weak to very strong correlations (*p*-values < 0.05) (**Supplementary Figure S8**).

### 3.10 Construction of *MMP14* co-expression prognostic model

**Figure 4:**
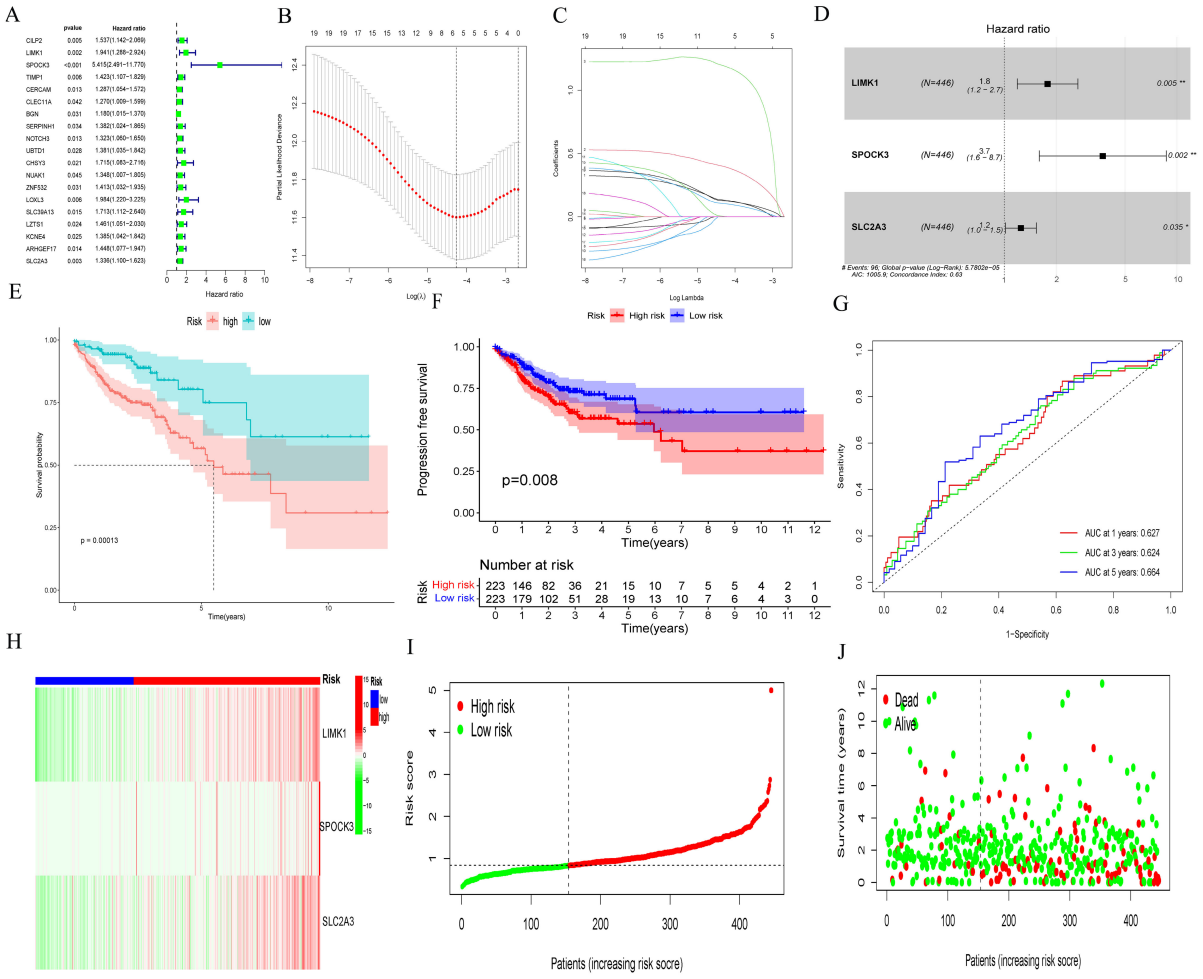
Development of prognostic gene markers based on MMP14-associated co-expression genes and MMP14. **(A)** Univariate Cox regression analysis to assess prognostic significance; **(B, C)** LASSO assays for feature selection; **(D)** Multivariate Cox regression analysis to construct prognostic model; **(E, F)** Evaluation of the impact of the prognostic model on OS and PFS; **(G)** ROC curves for model validation. **(H-J)** Heat map illustrating expression patterns of six genes, the distribution of risk score, and the survival status of CRC patients.

We employed univariate Cox regression analysis to identify 18 prognostic genes and evaluate their clinical significance in relation to *MMP14* co-expression in colorectal cancer. These genes include *CILP2, LIMK1, SPOCK3, TIMP1, CERCAM, CLEC11A, BGN, SERPINH1, NOTCH3, UBTD1, CHSY3, NUAK1, ZNF532, LOXL3, SLC39A13, LZTS1, KCNE4, ARHGEF17*, and *SLC2A3* (**Figure 4A**). Subsequently, we used LASSO regressions (**Figures 4B, C**) and multi-Cox regressions (**Figure 4D**) to refine the model genes. Risk assessment was completed using three genes - *LIMK1, SPOCK3*, and *SLC2A3* - each assigned coefficient of 0.5776808, 1.3139298, and 0.2217219, respectively. Using the TCGA-COAD cohort as a median, we divided the patients into two groups (high and low). A notable difference in OS and PFS was observed between the two groups (*p* = 0.00013, **Supplementary Figure S9E**), with the ROC curve indicating areas under 0.627, 0.624, and 0.664 for 1, 3, and 5 years, respectively (**Figure 4G**). CRC patients in the high-risk group exhibited higher levels of *LIMK1*, *SPOCK3*, and *SLC2A3* (**Supplementary Figure S9H**). The mortality increased in correlation with the risk assessment score (**Figures 4I, J**).

### 3.11 Independent validation of the prognostic signature

To examine the potential of the risk score as a standalone predictor of CRC prognosis, clinicopathological characteristics, the risk score were collected and analyzed through univariate and multivariate Cox regression analyses. The risk score was identified as an independent predictor of prognosis in univariate and multivariate Cox regression analyses (**Supplementary Figures S9A, B**). ROC analysis was conducted to evaluate the AUC values for the risk score. The AUC value for the risk score was determined as 0.665 (**Supplementary Figure S9C**). These findings indicate that the risk score holds promise as an independent predictive marker.

### 3.12 External validation of the prognostic model in the GSE39582 cohort

For external validation, the robustness of the prognostic model was verified using the GSE39582 dataset. Patients were stratified into two groups following the algorithm and median derived from the TCGA and COAD cohorts. Individuals in the high-risk group exhibited significantly worse overall survival than those in the low-risk group, which was consistent with the findings from TCGA (**Supplementary Figure S10A**). The distribution of risk scores, patient survival status, and heatmap for the three genes closely resembled those observed in the TCGA-COAD dataset (**Supplementary Figures S10B–D**).

### 3.13 Detection of *MMP14* expression

**Figure 5:**
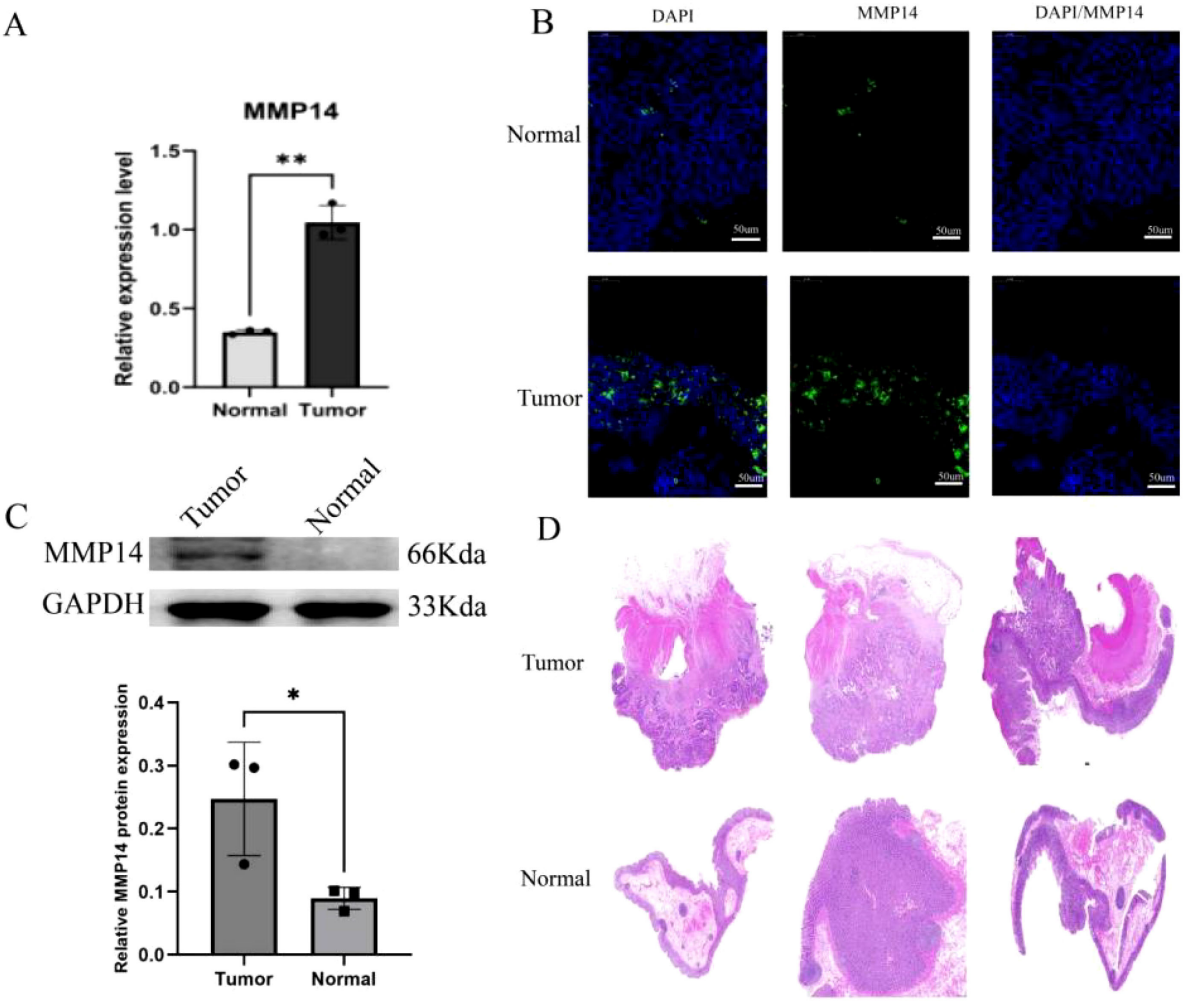
Experimental detection of MMP14 expression in clinical samples. **(A)** qRT-PCR for *MMP14* expression detection; **(B)** Western blot for detection of MMP14 expression; **(C)** Immunofluorescence for the detection of MMP14 expression, with DAPI staining for cellular localization; **(D)** H&E staining of three pairs of cancerous and adjacent tissues. Statistical significance is denoted as *P < 0.05, **P < 0.01.

To validate the results obtained from our initial analysis, we conducted qRT-PCR and Western blot assays. The findings demonstrated a significant increase in *MMP14* expression in tumor tissues compared to normal tissues (**Figures 5A, C**). Additionally, to enhance the reliability of these results, we employed immunofluorescence methods on a patient sample to observe MMP14 expression in cancer cells (**Figures 5B, D**). The findings suggested that tumor tissue samples exhibited a higher MMP14 fluorescent signal intensity than normal tissue samples.

### 3.14 Exploration of cell phenotypes after *MMP14* silencing

**Figure 6:**
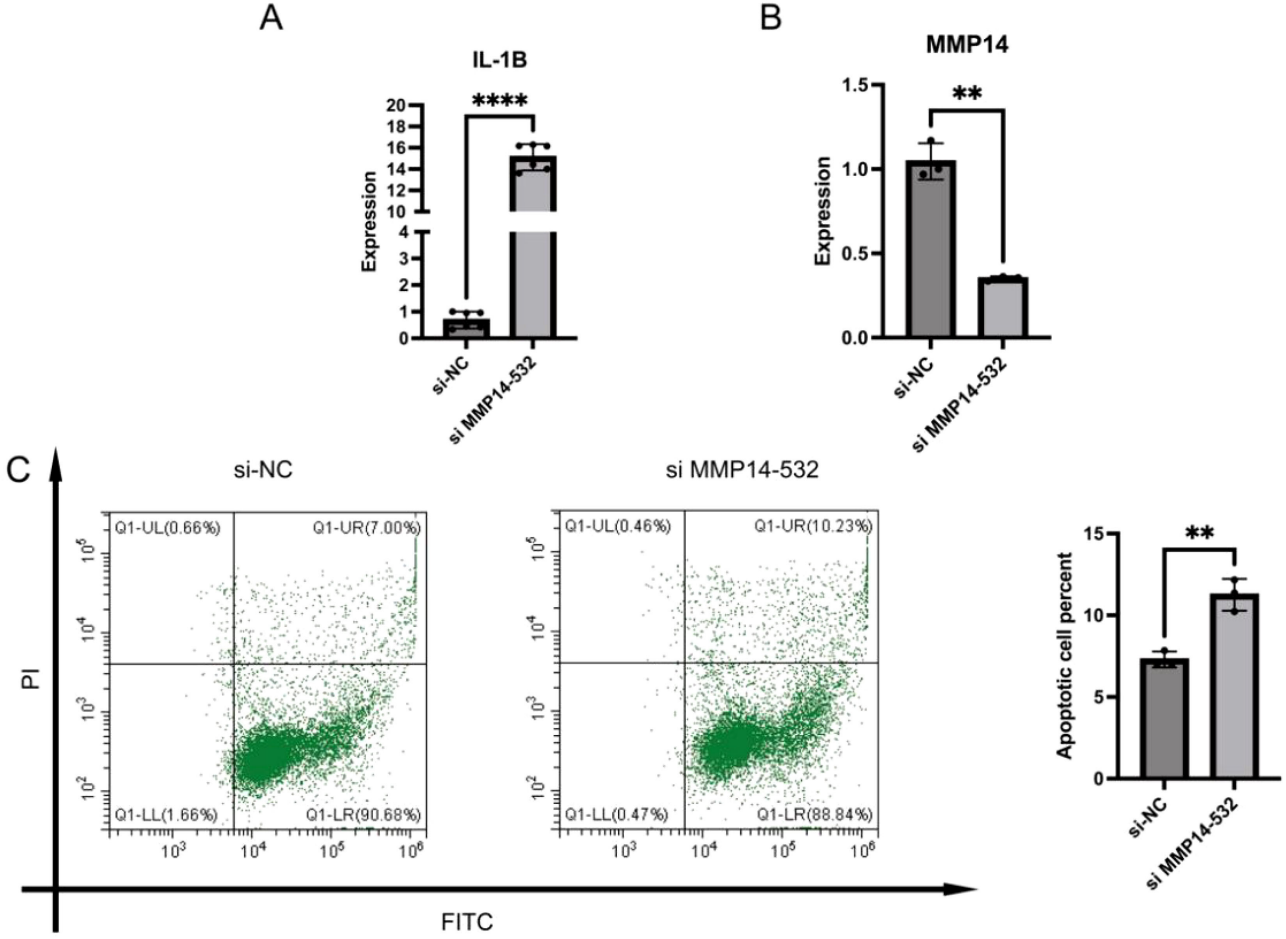
Construction of MMP14-silenced CRC cell system. **(A)** qRT-PCR detection of *IL-1*β mRNA expression; **(B)** Knockdown of MMP14 detected by qRT-PCR; **(C)** Flow cytometry was used to detect cell apoptosis following *MMP14* silencing. Statistical significance is denoted as **P < 0.01, and ****P < 0.0001.

To explore the function of *MMP14* in CRC, we developed an *MMP14*-silenced cell line. The success of our *MMP14* silencing approach was confirmed through qRT-PCR analysis (**Figure 6B**). Subsequently, we assessed cell apoptosis and observed a significant increase in the proportion of apoptotic cells when *MMP14* was silenced (**Figure 6C**). Additionally, the function of *MMP14* co-expressed genes was related to inflammatory response. The expression level of inflammatory factor *IL-1β* was measured using qRT-PCR, revealing a significant increase in its expression upon *MMP14* silencing (**Figure 6A**).

## 4 Discussion

Matrix metalloproteinases (MMPs), a group of fascinating genes, have been implicated in various processes such as invasion, cancer metastasis, immune surveillance evasion, and angiogenesis promotion (20). Among the MMPs, *MMP14* is essential in numerous biological processes observed in healthy and malignant cells (21). Despite its association with several cancer types, including lung cancer (22), gastric cancer (23, 24), breast cancer (BRCA) (25), pancreatic carcinoma (26), and bladder cancer (BLCA) (27), comprehensive analyses of *MMP14* in CRC remain limited (28).

The primary objective of this study was to elucidate the clinical significance and predictive value of MMP14 in CRC, including its comprehensive involvement in tumor-infiltrating immune cells. Using the TCGA database, we initially investigated *MMP14* expression levels, revealing a significant elevation in *MMP14* levels in CRC tissues compared to healthy tissues. This was further corroborated by Human Protein Atlas (HPA) data, which confirmed notably higher *MMP14* protein abundance in CRC compared to normal tissues. Moreover, we observed a meaningful correlation between *MMP14* mRNA levels and various clinical characteristics, including the TNM stage, suggesting that increased *MMP14* expression is a pivotal factor in CRC progression.

Furthermore, our analysis using the TCGA database revealed that *MMP14* was a significant indicator for OS and PFS. The results demonstrated that higher *MMP14* expression was associated with lower OS and PFS, aligning with previous findings by Yang et al. (29), who also reported a negative outcome associated with MMP14 expression. Notably, further analysis confirmed *MMP14* as a standalone prognostic indicator for overall survival.

We hypothesized that *MMP14* may be associated with immune cell infiltration in colorectal cancer due to its significant role in the tumor microenvironment (30). Our results corroborated this hypothesis, demonstrating a relationship between MMP14 levels and dendritic cells, macrophages (including macrophage M0, M1, and M2 subtypes), and neutrophils in COAD using TIMER and TIMER 2.0. Our study revealed a correlation between *MMP14* expression and various molecules regulating tumor immune cells, including CSF1R, HAVCR2, KDR, PDCD1LG2, TGFB1, CD70, CD86, and CXCL1. Additionally, CRC exhibited significantly elevated levels of resting CD4 memory T cells, macrophages M0 and M1, neutrophils, and other immune cells compared to normal tissue samples. Conversely, there were significantly fewer plasma cells and activated NK cells in CRC, suggesting a contributory role of immune cells in colorectal cancer progression, which aligns with previous research findings.

Moreover, the neutrophil-to-lymphocyte ratio is a valuable indicator for predicting adverse clinical outcomes and overall survival in CRC patients. This ratio reflects the presence of systemic inflammation and underscores the impact of neutrophils on disease progression (31). In addition, the mast cell-T cell axis has been implicated in influencing CRC growth in colitis-dependent and colitis-independent patients. Co-culture study has demonstrated that mast cell-primed tumors induce apoptosis in CRC cells (32).

GO and KEGG analyses were then conducted on co-expressed genes to explore potential pathways linked to *MMP14*, including proteoglycans in tumor development, Rap1 signaling pathway, phagosome, resistance to EGFR tyrosine kinase inhibitors, among others, shedding light on the complex mechanisms involving *MMP14*. Additionally, *MMP14* GSVA analysis in CRC uncovered a strong association between *MMP14* and pathways such as angiogenesis, TGFB signaling, ECM-related genes, and collagen formation, emphasizing the oncogenic role of *MMP14* in CRC progression.

Utilizing co-expression analysis, we constructed prognostic models and gained insights into the molecular mechanisms underlying *MMP14*, enhancing our understanding of its impact on disease prognosis. This facilitates more precise predictions and personalized treatment strategies for CRC patients. A prognostic model incorporating *MMP14* co-expression genes was developed, with risk scores calculated based on gene coefficients and expressions. These risk scores strongly correlate with colorectal cancer survival rates, encompassing overall survival and progression-free survival. These findings indicate the potential of *MMP14* to accurately identify individuals at high risk among CRC patients. However, the lack of validation datasets and *in vitro* and *in vivo* trials to explore the roles of *MMP14* limited our research. Further sophisticated studies are warranted to support our conclusions.

In conclusion, as a pro-oncogene, *MMP14* significantly contributes to the development of CRC and is associated with immune cell infiltration and poor prognosis. Therefore, *MMP14* can serve as a biomarker for predicting the prognosis of colorectal cancer patients.

## Data Availability

The original contributions presented in the study are included in the article/**Supplementary Material**. Further inquiries can be directed to the corresponding author.
