# Supplementary material for: Overexpression of MMP14 is associated with poor prognosis and immune cell infiltration in colon cancer
Source: Front Oncol. 2025 Apr 25;15:1564375. doi: 10.3389/fonc.2025.1564375 (PMC12062125; doi:10.3389/fonc.2025.1564375)
Supplement: Supplementary file 1 [file DataSheet1.pdf]

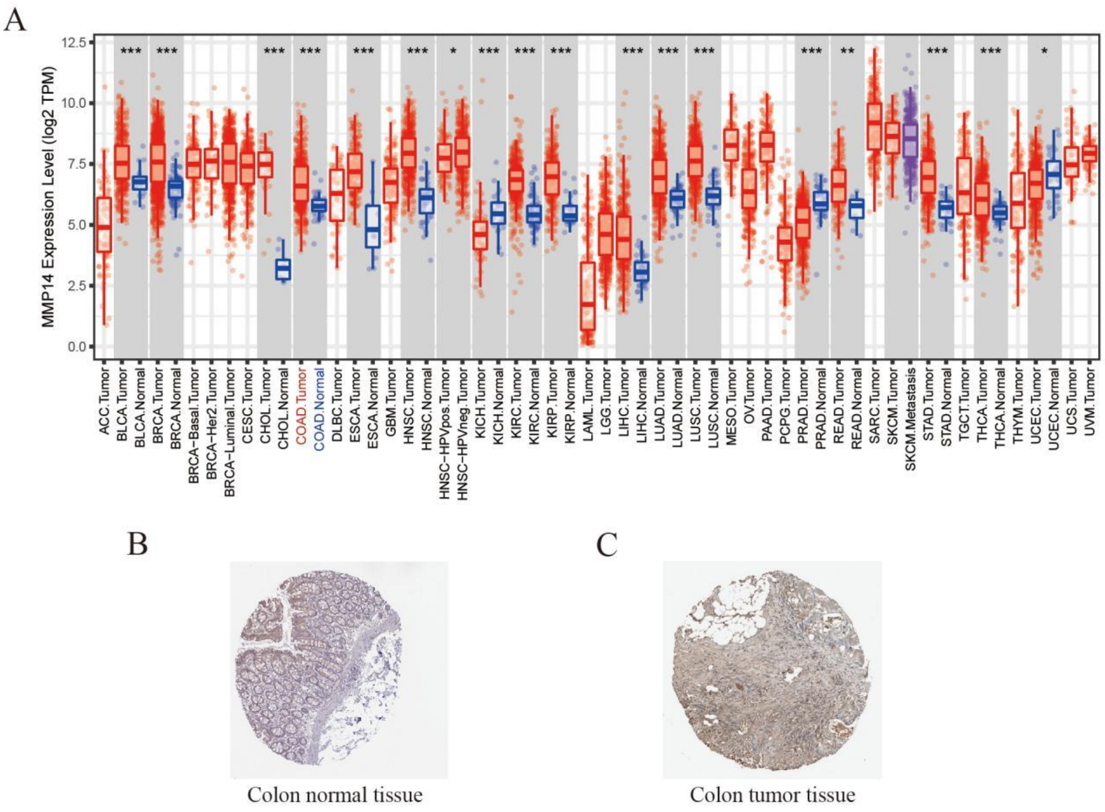

Figure S1. MMP14 expression in different databases. A. MMP14 expression in pan-cancer in TIMER (33 cancers); B. MMP14 protein expression in normal tissue; C. MMP14 protein expression in CRC tissue.

ACC, adrenocortical carcinoma; BLCA, bladder urothelial carcinoma; BRCA, breast invasive carcinoma; CESC, cervical squamous cell carcinoma and endocervical adenocarcinoma; CHOL; cholangiocarcinoma; COAD, colon adenocarcinoma; DLBC, lymphoid neoplasm diffuse large B-cell lymphoma; ESCA, esophageal carcinoma; GBM, glioblastoma multiforme; HNSC, head and neck squamous cell carcinoma; KICH, kidney chromophobe; KIRC, kidney renal clear cell carcinoma; KIRP, kidney renal papillary cell carcinoma; LAML, acute myeloid leukemia; LGG, lower brain grade glioma; LIHC, liver hepatocellular carcinoma; LUAD, lung adenocarcinoma; LUSC, lung squamous cell carcinoma; MESO, mesothelioma; OV, ovarian serous cystadenocarcinoma; PAAD,

pancreatic adenocarcinoma; PCPG, pheochromocytoma and paraganglioma; PRAD, prostate adenocarcinoma; READ, rectum adenocarcinoma; SARC, sarcoma; SKCM, skin cutaneous melanoma; STAD, stomach adenocarcinoma; TGCT, testicular germ cell tumors; THCA, thyroid carcinoma; THYM, thymoma; UCEC, uterine corpus endometrial carcinoma; UCS, uterine carcinosarcoma; UVM, uveal melanoma.

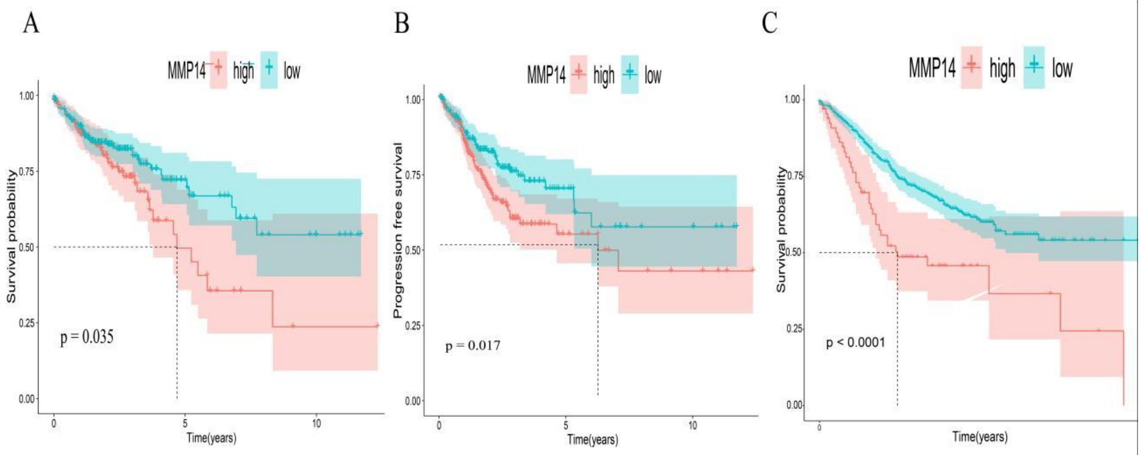

Figure S2. Overexpression of *MMP14* was strongly correlated with OS and PFS. A. Overall survival (OS) in TGCA-COAD; B. Progression-free survival (PFS) in TGCA-COAD; C. Overall survival (OS) in GSE39582.

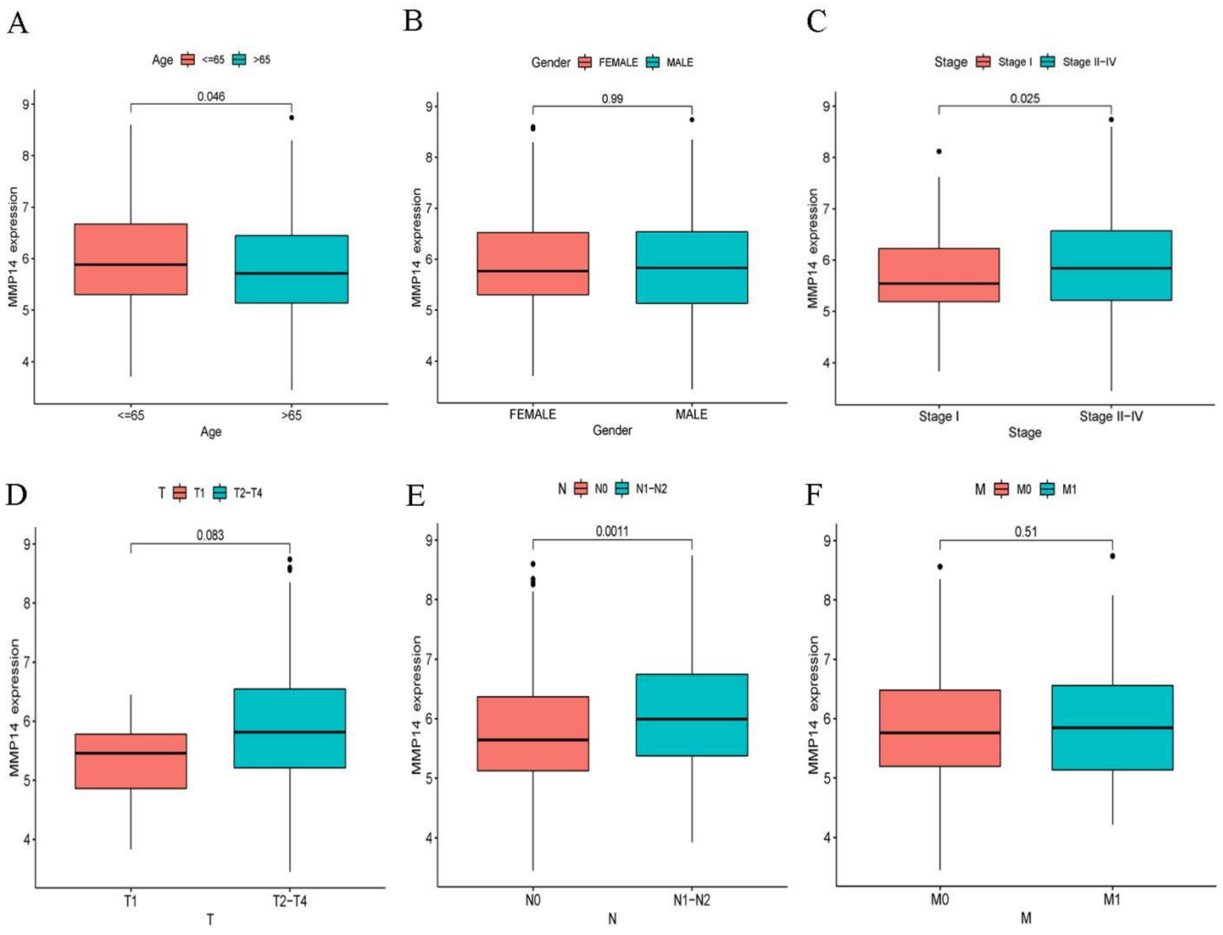

Figure S3. Correlation between MMP14 and clinical characteristics in CRC. A. age; B. gender; C. stage; D. T stage; E. N stage; F. M stage.

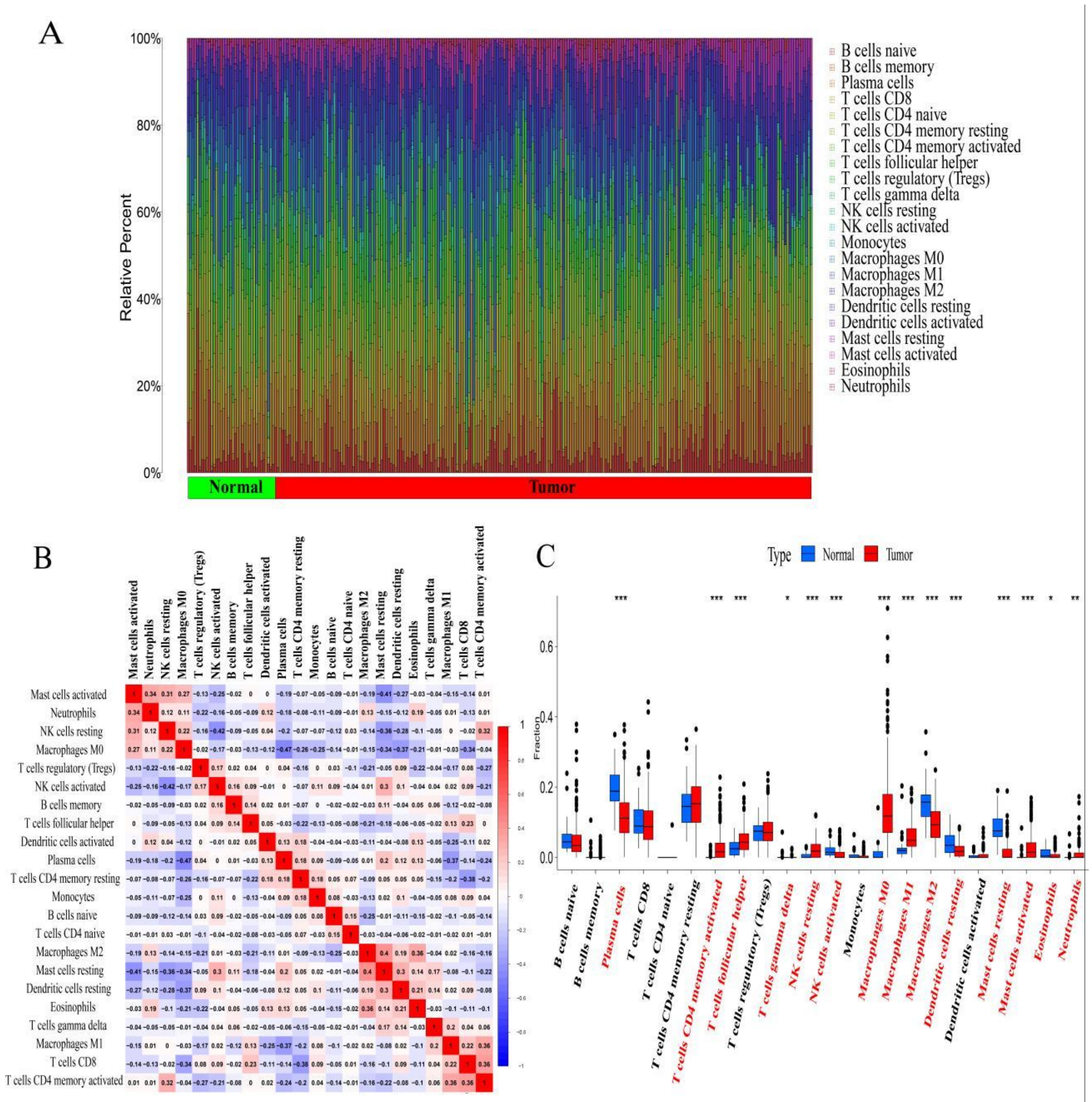

Figure S4. Analysis of the composition of 22 TIICs and their associations in CRC and normal samples. A. Heatmap of 22 TIICs and immune cells across TCGA-COAD (Tumor: 227 cases, Normal: 38 cases); B. Pearson correlation coefficient analyzed the matrix of 22 types of TIICs in CRC; C. Differences in the constitution of TIICs in normal and CRC specimens.

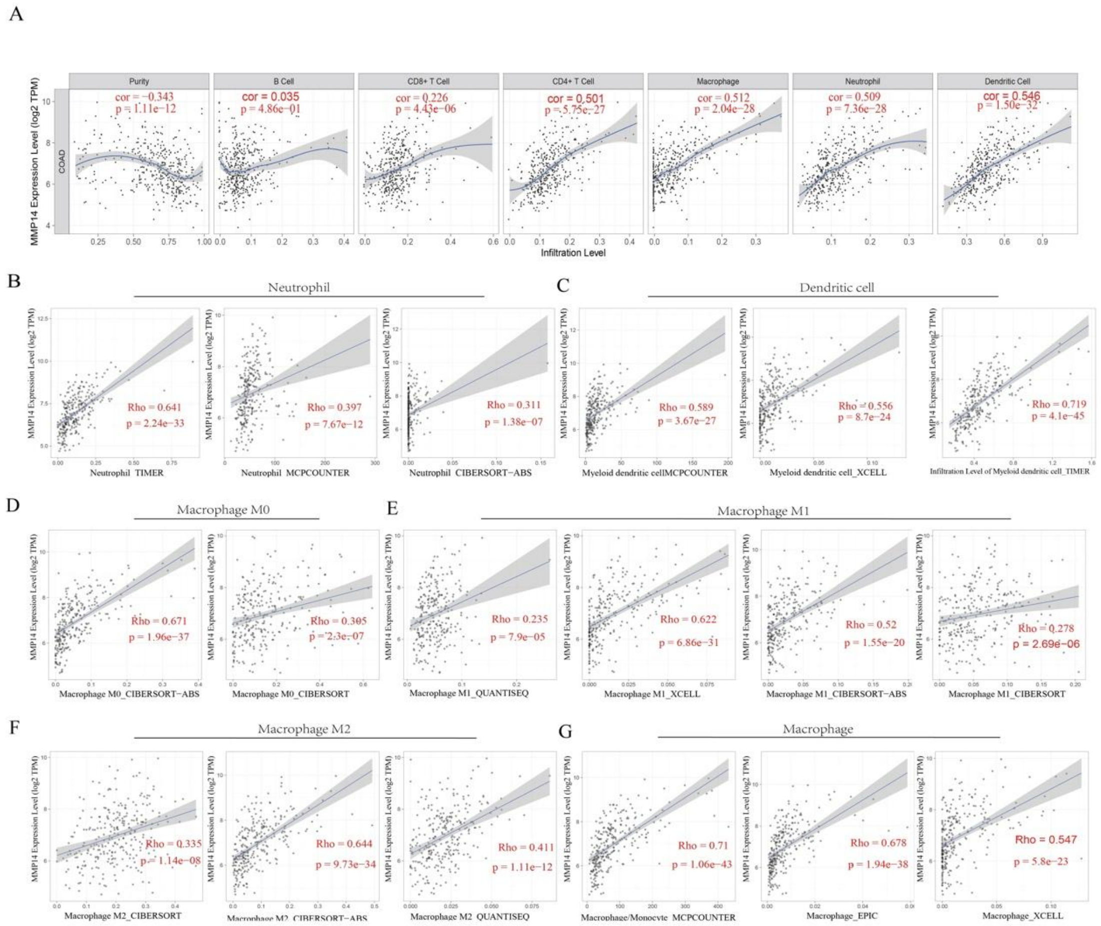

Figure S5. Correlation between MMP14 and immune infiltration cells in CRC. A. Correlation between MMP14 expression and immune cell infiltration level using TIMER; B. Correlation between MMP14 and immune cell infiltration level with different methods using TIMER 2.0.

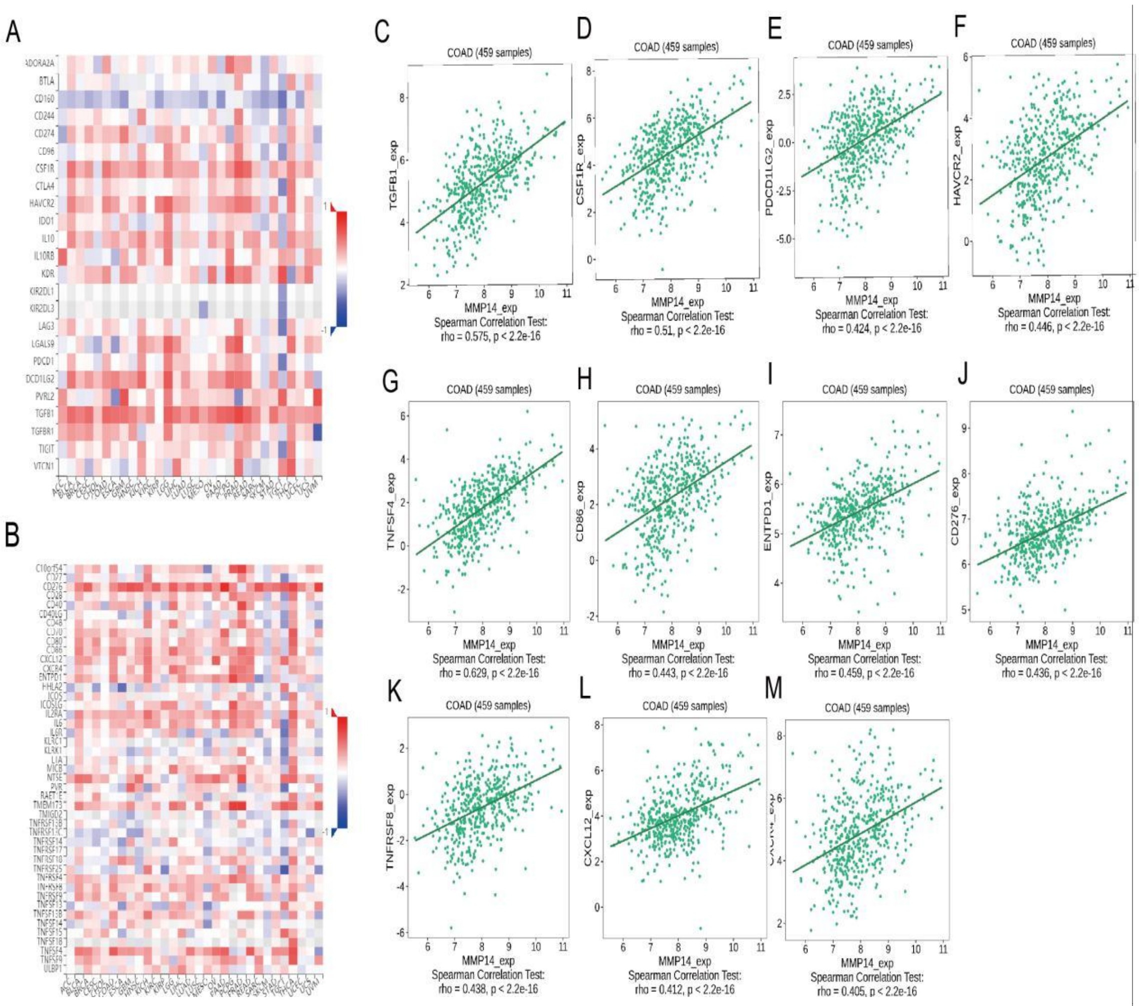

Figure S6. Correlation between *MMP14* and tumor immune cell regulatory molecules in CRC. A, B. Correlation heatmap showing the relationship between *MMP14* and representative immunoinhibitors and immunostimulators genes; C-F. Immunoinhibitors genes, including *TGFB1, CSF1R, PDCD1IG2, HAVCR2* displaying their correlation with MMP14; G-M. Immunostimulators genes, including *TNFSF4, CD86, ENTPD1, CD276, TNFRSF8, CXCL12*, and *CXCR4,* demonstrating their correlation with MMP14.

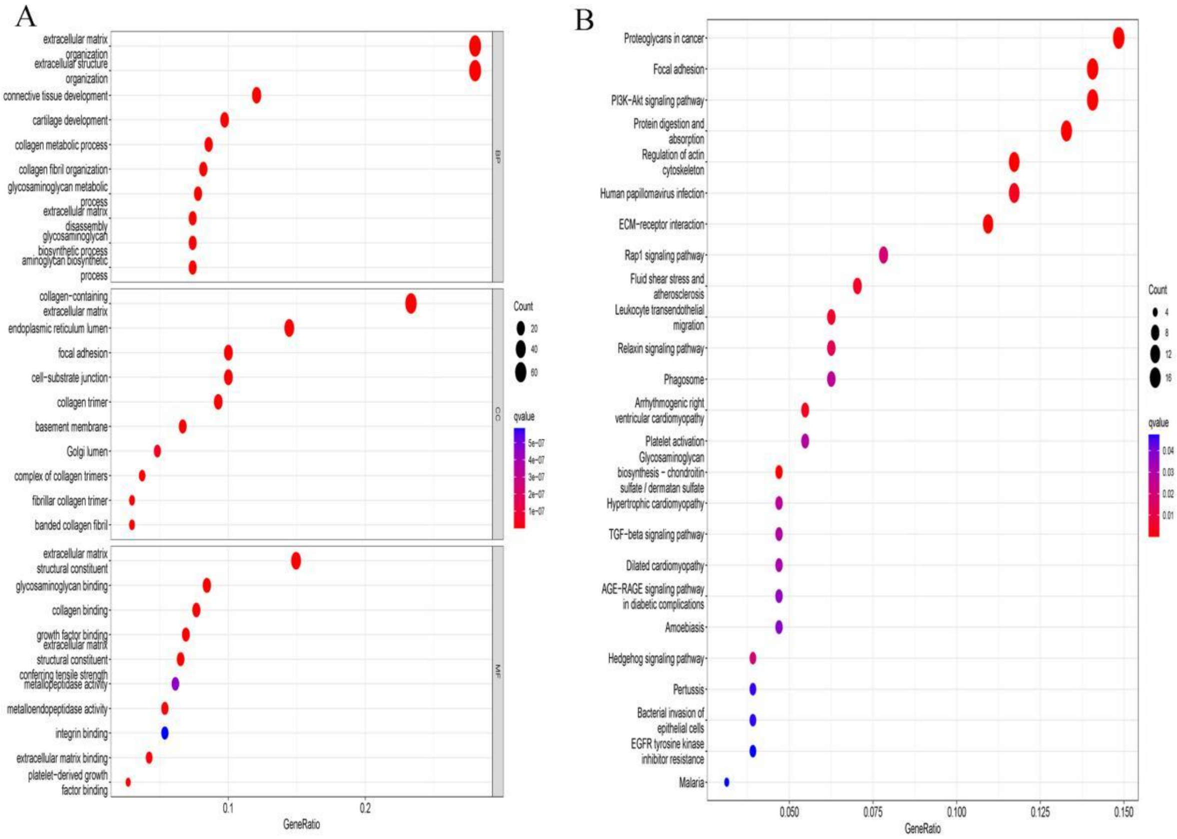

Figure S7. Functional enrichment analysis of co-expression genes of MMP14. A. GO analysis of co-expressed genes of MMP14; B. KEGG pathway enrichment assessment of co-expressed genes of MMP14.

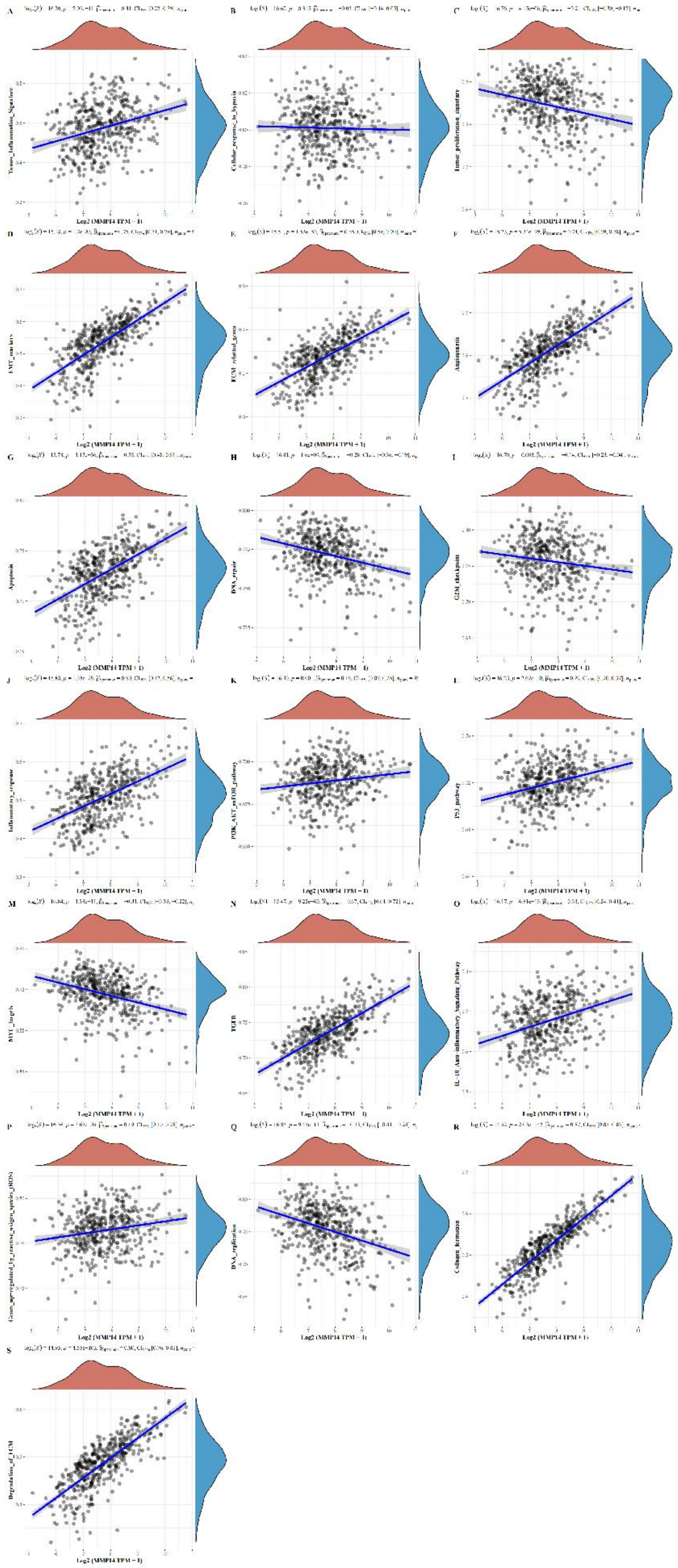
A
Tumor_Inflammation_Signature
B
Cellular_response_to_hypoxia
C
Tumor_proliferation_signature
D
EMT_markers
E
ECM_related_genes
F
Angiogenesis
G
Apoptosis
H
DNA_repair
I
G2M_checkpoint
J
Inflammatory_response
K
PI3K_AKT_mTOR_pathway
L
P53_pathway
M
MYC_targets
N
TGFB
O
IL_10_Anti-inflammatory_Signaling_Pathway
P
Genes_up-regulated_by_reactive_oxygen_species_(ROS)
Q
DNA_replication
R
Collagen_formation
S
Degradation_of_ECM
Log2 (MMP14 TPM + 1)

Figure S8. The analysis of MMP14-related gene set variation analysis (GSVA) results revealed a positive correlation between MMP14 expression and various biological processes.

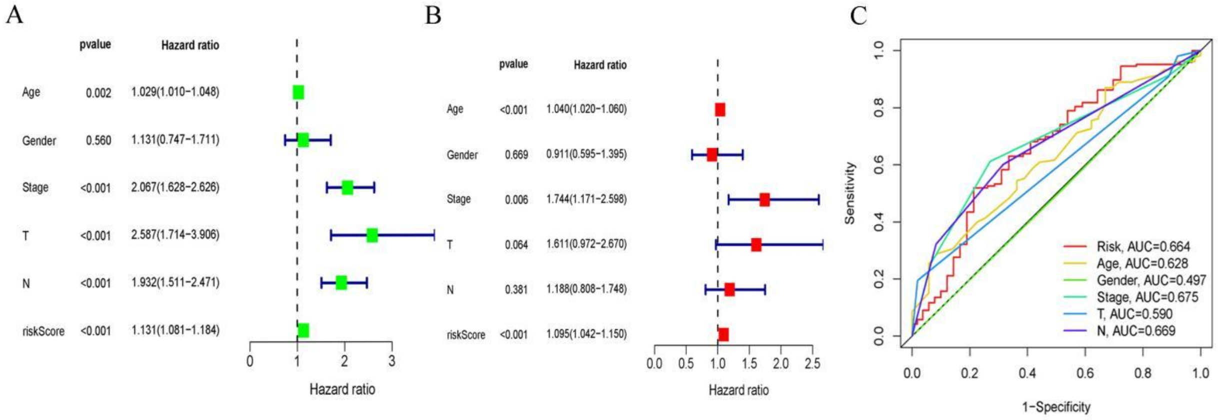

Figure S9. Verification of the universal applicability of the prognostic model. A, B. Univariate and multivariate regression analysis of the relationship between the risk score and clinicopathological characteristics; C. ROC analysis of the Risk score and clinicopathological characteristics.

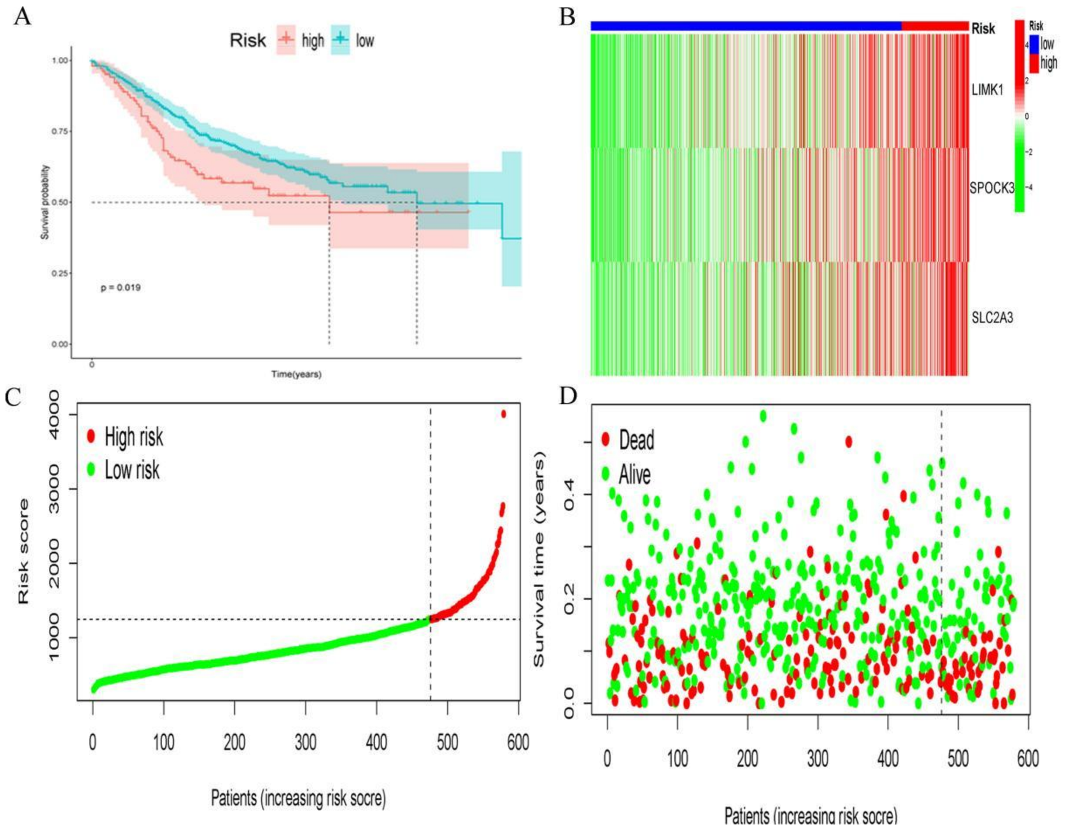

Figure S10. External validation of the prognostic model. A. OS analysis; B-D. Heatmap, distribution, and survival status.
